# Diseases of Women

**Published:** 1898-12

**Authors:** 


**Diseases of Women.** By J. C. WEBSTER, M.D. Pp. xxii., 688. Edinburgh: Young J. Pentland. 1898.

By a somewhat full consideration of allied points in embryology, anatomy, and comparative anatomy, Dr. Webster has ensured a scientific basis for his subject, and by this means he has succeeded in giving an unusual interest to gynæcology, though perhaps these matters have a somewhat disproportionate allowance to the general size of the book. To those interested, the appendix on the author's explanation of menstruation is, however, well worth reading. The work is carefully written, is up to date, and its teachings are sound and trustworthy. The illustrations are numerous and clear.

